# Case Report: Atypical acute compartment syndrome of the forearm in a child following minor trauma with consecutive osteomyelitis

**DOI:** 10.3389/fsurg.2024.1370558

**Published:** 2024-05-15

**Authors:** J. Everaert, A. Delafontaine, J. Juanos Cabanas, G. Leclercq, H. Jennart, B. Baillon

**Affiliations:** ^1^ Orthopedics and Traumatology Department, Erasme Hospital, Brussels, Belgium; ^2^ Laboratoire D'Anatomie Fonctionnelle, Faculté des Sciences de la Motricité, Université Libre de Bruxelles, Bruxelles, Belgium; ^3^ Laboratoire d’Anatomie, de Biomécanique et d’Organogenèse, Faculté de Médecine, Université Libre de Bruxelles, Bruxelles, Belgium; ^4^ Orthopedics and Traumatology Department, Tivoli University Hospital, La Louvière, Belgium; ^5^ Orthopedics and Traumatology Department, IRIS SUD Hospitals, Forest, Belgium

**Keywords:** pediatric, trauma, acute compartment syndrome, infection, osteomyelitis

## Abstract

**Introduction:**

Forearm compartment syndrome (CS) in children is above all a clinical diagnosis whose main cause is traumatic. However, rarer causes such as infection can alter its clinical presentation.

**Clinical case:**

An 8-year-old boy has been seen in the emergency department complaining of severe forearm pain under a splint in a mild traumatic context. The previous radiological imaging examination three days before had not revealed any fractures. On admission, he presented with major signs of skin inflammation, loss of mobility, paresthesia and a significant biological inflammatory syndrome. The acute CS diagnosis has been made and was treated, but its atypical presentation raised a series of etiological hypotheses, in particular infectious, even if it remains rare. Complementary imaging examinations confirmed the presence of osteomyelitis of the distal radius as well as an occult Salter-Harris II fracture.

**Discussion:**

Beyond the classic “five P's of CS” -pain, paresthesia, paralysis, pallor and pulselessness-, CS's clinical presentations are multiple, especially in pediatric patients. In children, severe pain and increasing analgesic requirement must be indicators of a CS. We hypothesize that this patient sustained a nondisplaced Salter-Harris II fracture with a hematoma colonized by hematogenous osteomyelitis explaining its initial clinical presentation.

**Conclusion:**

Hematogenous osteomyelitis complicated by CS is rare and may be accompanied by a traumatic history. It's atypical presentation in pediatric patients requires vigilance and prompt diagnosis given the disastrous and irreversible complications.

## Introduction

Compartment syndrome (CS) is characterized by elevated intra-compartmental pressure within a non-expansile muscle compartment, leading to vascular compromise, progressing towards ischemia and tissue necrosis. It is a serious and rare condition in children, primarily diagnosed clinically (1) and could be confirmed by needle manometry (2).

Concerning CS diagnosis in children younger than 10 years, the usual causes are vascular injury or infection. Conversely, in children older than 14 years, trauma or surgical positioning typically underlie the condition (3).

While the traditional criteria for diagnosing compartment syndrome in adults include the five Ps (pain out of proportion or increasing in severity, pain with passive stretch, palpable tenseness, paresthesia, and paralysis/motor weakness), a mnemonic of three As has been proposed for children (4). These indicators consist of increasing anxiety, escalating agitation, and heightened analgesic needs. It could be very helpful for clinicians for establish earlier CS diagnosis, notably in children with silent hand CS form including atypical symptoms (5).

Although there's frequently a significant delay between injury, diagnosis and the necessity for fasciotomy in cases of compartment syndrome, the majority of children still attain favorable results (6).

Etiologies include bone trauma, iatrogenic causes (restraint, infiltration on intravenous catheters), intra-compartmental hemorrhages, and, rarely, infection.

Delayed diagnosis results in irreversible damage to the musculoskeletal and nervous systems (3). It must be notified that in the literature there is still no evident threshold of compartment pressure in children which present upper normal compartment pressure compared to adults, notably for the lower limb (2). Clear surgical indications are only available for adults (7).

The atypical presentation of CS in a pediatric patient at our institution prompts an investigation into its etiology.

## Case description

**Figure 1:**
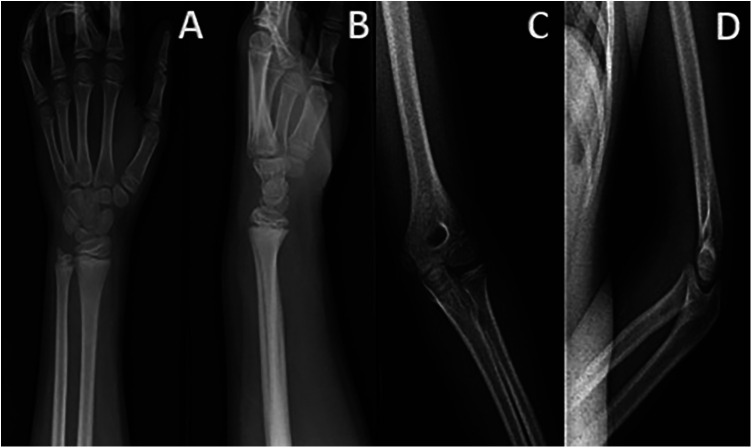
Frontal and lateral radiographs of the left wrist (**A**–**B**) and elbow (**C**–**D**) without evidence of bone lesions or signs of joint effusion.

An 8-year-old boy presented to the emergency department with left wrist pain following a minor fall. His history included atopic dermatitis with associated with excoriations due to scratching behavior. Pain radiated from the wrist to the elbow, accompanied by swelling, functional impairment, and abrasions. Standard radiographs were normal (Figure 1). The diagnosis of wrist contusion with suspected sprain was made, and he was treated with a brachio-antebrachial plaster cast and analgesics.

Three days later, the child was readmitted due to worsening pain unresponsive to first-line analgesics, lethargy, and hand paresthesia. He was afebrile, with a swollen, erythematous, warm forearm and generalized induration. Pain was exacerbated by passive mobilization. Blood work revealed leukocytosis (20.71 × 10^3/mm3^), elevated neutrophils (17.09 × 10^3/mm3^), and C Reactive Protein (174.3 mg/L). Blood cultures were obtained, and repeat radiographs were negative. Doppler ultrasound and joint aspiration yielded no diagnostic clues.

Persistent pain despite intravenous opioid analgesia raised suspicion of CS. Immediate surgical intervention confirmed the diagnosis using the Whitesides technique (8), with compartment pressures two to three times higher than normal (30 mmHg) and contralateral pressures. Volar compartment fasciotomy revealed extrusion of macroscopically normal muscle masses. Dorsal compartment pressures normalized post-incision, and no further compartments were opened.

**Figure 2:**
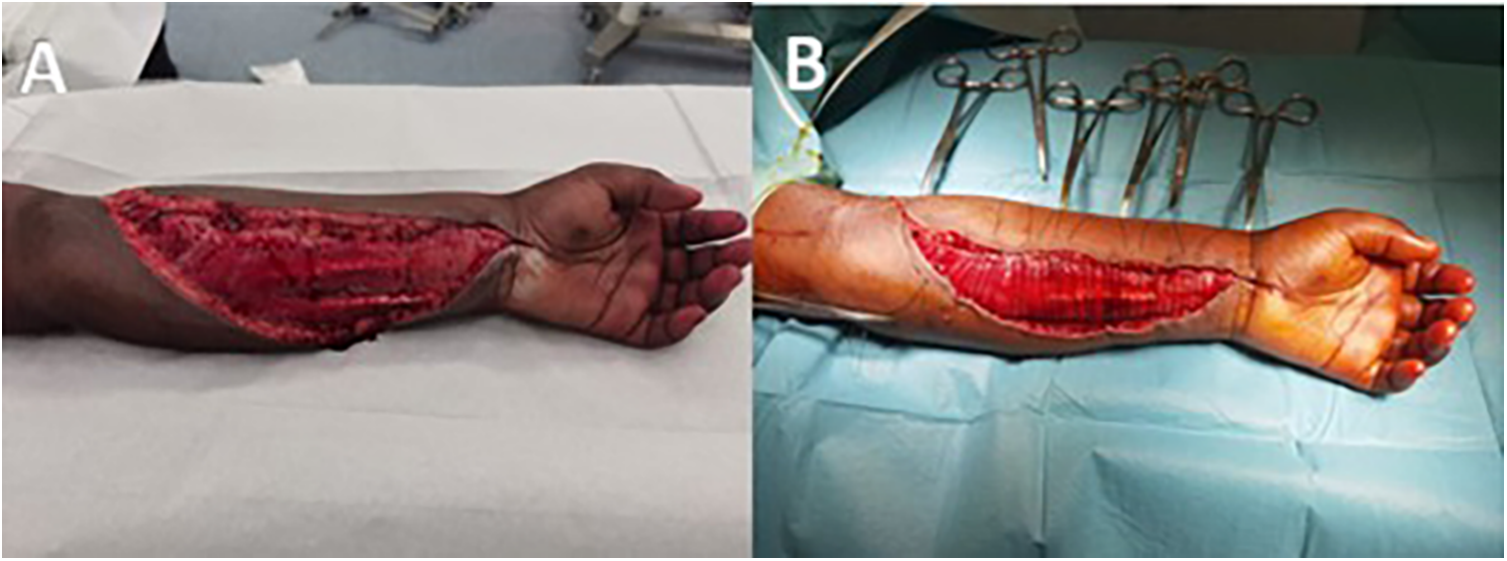
Preoperative (**A**) and postoperative (**B**) photographs of the “second look”.

Empirical intravenous Cefuroxime (1,500 mg ×4/day) was administered, switched to Cefazolin (1,500 mg × 4/day) on day 1 due to fever. The patient underwent early physiotherapy and wound care under anesthesia three times per week. By day 2, clinical improvement was observed, allowing progressive closure (Figure 2). Inflammatory markers decreased, and blood cultures were negative.

**Figure 3:**
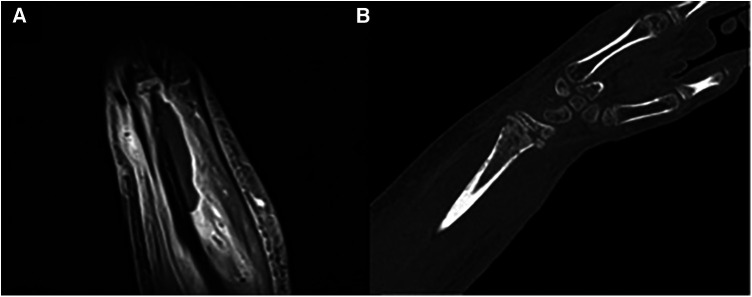
Deep abscess in contact with the periosteum of the distal end of the radius (**A**), altered, indicative of osteomyelitis (magnetic resonance imagery with gadolinium—T2 coronal section) and salter-harris type II fracture of the distal radius and osteolytic gaps (**B**) in the metaphyseal area (CT scan).

Despite persistent pain and the inability to close the wound, a magnetic resonance imagery with gadolinium on day 12 revealed osteomyelitis (Figure 3A). A subsequent computed tomography scan confirmed a Salter 2 distal radius fracture (Figure 3B).

**Figure 4:**
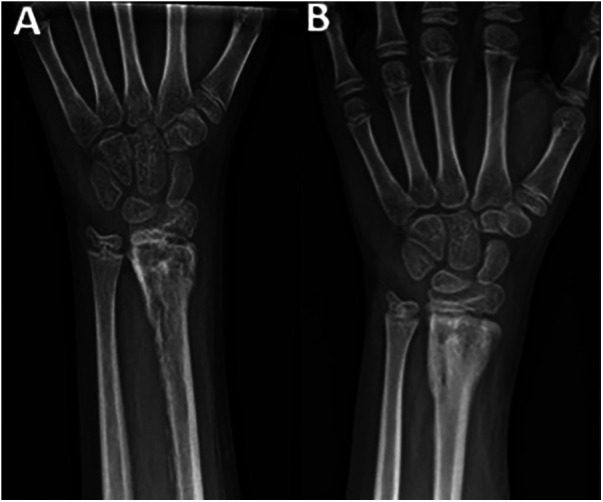
Follow-up frontal radiographs of the left wrist on the 38th (**A**) and 73rd (**B**) day postoperative.

Abscess drainage was performed, identifying multi-sensitive Staphylococcus aureus. Progressive closure proceeded smoothly. Clinical and laboratory outcomes were favorable. Intravenous antibiotics continued for 27 days, followed by oral Sulfamethoxazole-trimethoprim for six weeks. The patient was discharged with a palmar splint and active-passive finger physiotherapy. At 9 weeks, clinical examination revealed nearly complete joint recovery, absence of pain and neurological symptoms, and bone consolidation on radiographs (Figure 4).

## Discussion

CS in children is a rare condition with challenging diagnosis, particularly due to non-verbal complaints and child obtundation (9). Bae et al. (1) demonstrated low sensitivity of typical clinical signs (pain, paresthesia, paralysis, pallor, and pulselessness) (1). Pain remains the major diagnostic element, especially if well-managed analgesia fails (1, 3). Multifactorial delays in diagnosis can occur, particularly without traumatic conditions (3).

In our case, the clinical presentation initially suggested an infectious etiology, considering abrasions and excoriations. However, the traumatic context, immobilization, opioid-resistant pain, and neurological symptoms prompted compartment pressure measurement, facilitating rapid diagnosis and intervention for CS. We measured the pressure in the contralateral “healthy” forearm compartment to ensure that our measurement method did not produce aberrant values (i.e., validated the setup on the anesthesiologist's arterial line) considering the unusual clinical history and the absence of major trauma explaining the diagnostic hypothesis.

Childhood osteoarticular infections are common, with acute osteomyelitis (AO) incidence ranging from 2 to 13 per 100,000 children in industrialized countries (10). Staphylococcus aureus is a predominant causative agent (10, 11). Symptoms typically include swelling, redness, warmth, pain, and variably, fever (11). According to Pääkkönen, up to one in five cases of AO follows trauma within two weeks (12).

Similar to other authors (13, 14), we propose that our patient had an occult fracture, where hematoma secondary to hematogenous colonization became infected. Infection induced tissue inflammation and reorganization, coupled with increased intra-compartmental pressure leading to CS.

The possibility of iatrogenic inoculation during the first surgical intervention cannot be ruled out, but it does not explain the initial infectious clinical presentation and seems less likely.

During the initial fasciotomy, the dissection did not extend to periosteum in the deep volar compartment. Therefore, we did not observe any pre-organized collections [regarding what was observed by Mulcahey et al. (13) and Shaw et al. (14)]. We cannot definitively exclude it, but it is highly likely that the hematogenous infectious process was already underway given the infectious clinical presentation. This was also not observed on preoperative ultrasound, which remained unreliable due to being performed by an inexperienced resident in an emergency setting. In this context, the use of emergency magnetic resonance imagery protocol for pediatric osteoarticular infection could be very useful for accelerate the process, abstain from sedation and enhance the quality of images without the need for contrast (15).

Indeed, there is also a risk of osseous bridging at the distal physis of the radius, which is monitored by radiography to ensure there are no complications (i.e., such as radiocarpal conflict due to axial deviation) and to determine if corrective surgical intervention will be necessary.

## Conclusion

This case highlights the diverse clinical presentations of pediatric CS and underscores its numerous etiologies. Hematogenous AO is rare and may present atypically, especially in the pediatric population, necessitating vigilance due to potential irreversible complications.

## Data Availability

The original contributions presented in the study are included in the article/Supplementary Material, further inquiries can be directed to the corresponding author.
